# The Moderating Effect of Self-Reported State and Trait Anxiety on the Late Positive Potential to Emotional Faces in 6–11-Year-Old Children

**DOI:** 10.3389/fpsyg.2018.00125

**Published:** 2018-02-20

**Authors:** Georgia Chronaki, Samantha J. Broyd, Matthew Garner, Nicholas Benikos, Margaret J. J. Thompson, Edmund J. S. Sonuga-Barke, Julie A. Hadwin

**Affiliations:** ^1^Developmental Cognitive Neuroscience (DCN) Laboratory, University of Central Lancashire, Preston, United Kingdom; ^2^Division of Neuroscience and Experimental Psychology, University of Manchester, Manchester, United Kingdom; ^3^School of Psychology, University of Southampton, Southampton, United Kingdom; ^4^School of Psychology, University of Wollongong, Wollongong, NSW, Australia; ^5^Department of Cognitive Science, Macquarie University, Sydney, NSW, Australia; ^6^King’s College London, London, United Kingdom

**Keywords:** emotion, faces, LPP, anxiety, depression, children

## Abstract

**Introduction:** The emergence of anxiety during childhood is accompanied by the development of attentional biases to threat. However, the neural mechanisms underlying these biases are poorly understood. In addition, previous research has not examined whether state and trait anxiety are independently associated with threat-related biases.

**Methods:** We compared ERP waveforms during the processing of emotional faces in a population sample of 58 6–11-year-olds who completed self-reported measures of trait and state anxiety and depression.

**Results:** The results showed that the P1 was larger to angry than neutral faces in the left hemisphere, though early components (P1, N170) were not strongly associated with child anxiety or depression. In contrast, Late Positive Potential (LPP) amplitudes to angry (vs. neutral) faces were significantly and positively associated with symptoms of anxiety/depression. In addition, the difference between LPPs for angry (vs. neutral) faces was independently associated with state and trait anxiety symptoms.

**Discussion:** The results showed that neural responses to facial emotion in children with elevated symptoms of anxiety and depression were most evident at later processing stages characterized as evaluative and effortful. The findings support cognitive models of threat perception in anxiety and indicate that trait elements of anxiety and more transitory fluctuations in anxious affect are important in understanding individual variation in the neural response to threat in late childhood.

## Introduction

Understanding emotions from facial expressions plays an important role in children’s socio-emotional competence (Trentacosta and Fine, 2010). Recent studies have focused on associations between emotion processing of threatening (vs. neutral) stimuli and experiences of anxious affect in children and adolescents (review by Dudeney et al., 2015). Developmental research has been guided by conceptual models of attention and anxiety which have proposed that attention biases for threat stimuli are evident in early (automatic) information processing stages and these processes cause or maintain anxious affect (e.g., Bar-Haim et al., 2007). While some theoretical frameworks suggest that information processing is a function of *current* emotional state such as state anxiety (e.g., Bower, 1981); most have argued that positive associations between anxious affect and attention to threat reflects elevated trait anxiety that interacts with state anxiety (Mogg and Bradley, 1998, 2016; Bar-Haim et al., 2007). In support, studies have found positive associations between self-reported trait anxiety symptoms in a community sample of children aged 9–12 years and reactions times to probes that followed angry vs. neutral faces, indicating an attentional bias for threat in late childhood (Waters et al., 2010). Similar results have been shown for children aged 5–13 years diagnosed with social phobia (Waters et al., 2011). Furthermore, in an eye movement task children aged 11–12 years diagnosed with pediatric anxiety disorder showed shorter saccade latencies to angry faces, compared to a healthy age-matched control group (Mueller et al., 2012).

Although theoretical models have suggested that processing biases to threat are best understood in the context of elevated symptoms of trait and state anxiety (e.g., Williams et al., 1997), few studies have directly compared the independent effect of trait anxiety, state anxiety, and depression symptoms on emotion processing biases. Concurrent state anxiety has been argued to exacerbate the effects of trait anxiety in an interactive way (Broadbent and Broadbent, 1988; Farrin et al., 2003). Some studies have shown that state anxiety is associated with attentional biases to threat only in individuals with high trait anxiety (Egloff and Hock, 2001), while the results of other studies suggest that state anxiety and trait anxiety contribute independently to attentional biases (Mogg et al., 1990). It remains unclear how state and trait anxiety symptoms contribute to threat-related attentional biases in children.

While behavioral and eye movement data have provided some support for cognitive models of anxiety, event-related potential (ERP) paradigms allow a clearer analysis of the time course associated with the processing of emotional stimuli and the identification of neural markers of anxiety-related attentional processes in children and adolescents. Developmental research has found that the P1 (reflecting early sensory processing) has been observed over parietal-occipital sites around 190 ms in response to happy and fearful faces early in development (Nelson and de Haan, 1996; Taylor et al., 2004). The N170 is an occipitotemporal negative potential occurring at 170 ms post stimulus onset linked to sensitivity in processing information from human faces (Bentin and Carmel, 2002). Research has shown larger N170 amplitudes for negative (i.e., angry) compared to positive (i.e., happy) and neutral faces in 14- to 15-year-olds (Batty and Taylor, 2006). In addition, the Late Positive Potential (LPP) is a positive parietal-occipital component that is evident from around 300 ms and that is proposed to signify elaborative or effortful processing of emotional stimuli (Schupp et al., 2000; Hajcak et al., 2010). The LPP has been found to be sensitive to the emotional content of human faces from the 1st months of life (Leppänen et al., 2007) and its amplitude is larger to positive and negative (compared to neutral) stimuli in 5–8-year-olds (Hajcak and Dennis, 2009; Solomon et al., 2012). Consistent with adult research, the LPP was larger following angry (vs. happy) faces in 7-year-old children (Kestenbaum and Nelson, 1992) and larger for sad compared to neutral faces at occipital sites in a passive viewing paradigm in 6-year-olds (Kujawa et al., 2012a). The LPP is also elicited in adults by non-affective, but task relevant, stimuli that require effortful cognitive processing (Matsuda and Nittono, 2015). Some studies using facial stimuli have shown small emotion effects on LPP amplitudes in children (Kujawa et al., 2012a,b). Similar research has found greater LPP amplitudes for pictorial stimuli (i.e., negative and positive images) compared with faces in children and adolescents (Kujawa et al., 2012b).

Considering the moderating effect of anxiety on ERP components, research supports the proposition that individuals with increased anxious affect allocate attention to threat at early processing stages. For example, a community sample of adult individuals with high trait anxiety showed increased P2 amplitudes and faster latencies in the occipital region when viewing centrally presented angry faces (Bar-Haim et al., 2005). In addition, O’Toole et al. (2013) found that increased N170 amplitudes to angry (vs. happy) faces in young children predicted the development of anxiety symptoms 2 years later. Further studies indicate that later ERP components (i.e., the LPP) are also potential neural markers of increased attention to threat (MacNamara and Hajcak, 2010). A study using an emotional flanker task, for example, found enhanced LPPs to angry (vs. happy) target faces in non-referred high socially anxious adults (Moser et al., 2008). A recent study showed that increased processing of unpleasant compared to neutral pictures (reflected by the posterior LPP) was associated with parent-rated child anxiety in a community sample of 5–7-year olds (DeCicco et al., 2012). However, this study used pictorial stimuli in a reappraisal task and has not examined trait and state anxiety and depression. A second study, using a passive viewing paradigm found that the degree to which unpleasant compared to neutral pictures elicited larger late anterior LPPs was associated with inhibited and fearful behavior in a community sample of 5–7-year olds (Solomon et al., 2012). Recently, Kujawa et al. (2015) also found enhanced LPPs to angry and fearful faces during an emotional face-matching task in 7–19-year-old adolescents diagnosed with social anxiety, separation anxiety and generalized anxiety disorders (compared to controls). This study, however, included a limited range of depressive symptoms and did not measure state anxiety.

Whereas early components (e.g., P1) are thought to be a marker of relatively automatic attention to emotional stimuli, later components (e.g., LPP) are argued to reflect more deliberate processing and allocation of attention to emotional stimuli (Foti and Hajcak, 2008). It has been suggested that the greater deployment of attentional processing resources to emotionally salient stimuli may occur due to feedback from the amygdala to visual cortical areas (Pessoa et al., 2002; Amaral et al., 2003; Vuilleumier et al., 2004). Early components (e.g., P1 and N170) represent a useful measure of preferential processing of threat. In anxious individuals threatening (e.g., angry) faces rapidly and automatically heighten awareness and recruit attentional resources (Eysenck, 1992; Hadwin et al., 2009; Hadwin and Field, 2010). Late positivities (e.g., LPP) may indicate more deliberate processing based on the elaborated meaning of facial stimuli (Hajcak et al., 2010). Research has shown that the LPP is sensitive to emotion regulation strategies such as directed reappraisal (Foti and Hajcak, 2008; MacNamara et al., 2016). The LPP is modulated by a brain network composed of cortical and subcortical structures, such as the amygdala, associated with visual and emotional processing (Liu et al., 2012). Research examining reappraisal and the LPP suggests that children are able to effectively use reappraisal to modulate how they process unpleasant emotional stimuli, as measured via the LPP, and that changes in the LPP are associated with individual differences in mood and anxiety (Dennis and Hajcak, 2009). These models have suggested that increased elaborate processing in response to unpleasant stimuli (as indicated by increasing amplitude of the LPP) may index enhanced attention to negative information in anxiety whereas decreased elaborative processing of unpleasant stimuli (as indicated by decreased amplitude of the LPP) may reflect a mechanism of avoidance of threat (Weinberg and Hajcak, 2011). Based on this model, individual differences in attention to threat may reflect a propensity to maintain or develop symptoms of anxiety in children. Identifying a biomarker for this type of biased processing (e.g., P1 and N170) can help us understand the development of anxiety and identify ‘at risk’ individuals.

While there is an emerging picture in developmental research highlighting the role of individual differences in both negative affect and the processing of emotional stimuli, research has not explored associations between state and trait anxiety symptoms on ERPs to facial emotion processing, despite evidence from adult studies showing larger LPP amplitudes to unpleasant compared to neutral stimuli in individuals with higher state anxiety (MacNamara and Hajcak, 2009). Similar research with adults has further shown that individual differences in state anxiety moderate the amygdala response to fearful faces (Bishop et al., 2004). It is not clear whether neural alterations underlying children’s processing of threatening information are associated with enduring personality characteristics (trait anxiety) or more temporary anxious state regardless of the personality trait (or their interaction).

Recent studies in adults have aimed to examine the differential effects of anxiety and depression symptoms on the LPP to emotional stimuli. Research has shown that anxiety and depression may have opposing associations with the LPP; while anxiety was associated with *enhanced* LPP to threat, depression was linked to *reduced* LPP to threat (MacNamara et al., 2016). For example, an increased number of self-reported depressive symptoms was associated with reduced LPPs to angry faces in a group of 7–19-year-olds diagnosed with an anxiety disorder during an emotional face-matching task (Kujawa et al., 2015). The finding of a blunted/reduced emotional response (as reflected by the LPP) is consistent with theories suggesting disengagement from emotional stimuli more generally in depression (Proudfit et al., 2015). The findings are also consistent with results from behavioral studies which have found that anxiety and not depression is characterized by increased attention to threat (e.g., Hadwin et al., 2003; Waters et al., 2010).

The present study aimed to extend previous research to explore associations between child self-reported anxiety and depression symptoms and the processing of threat (angry faces), and positive (happy faces) vs. neutral (neutral faces) stimuli measuring ERP responses. It considered whether links between early and late ERPs to emotional information are associated with elevated state and trait anxiety symptoms (and their interaction) in young children. In particular, we investigated whether trait anxiety and depression symptoms would explain variance in ERP amplitude to angry vs. neutral faces above and beyond that explained by state anxiety. We included self-reported data on children’s anxiety and depression, because parents have been shown to be relatively poor at reporting accurately on their child’s internalizing symptoms (Choudhury et al., 2003). We used facial stimuli because human faces represent unique social signals that elicit differential ERP responses (Kujawa et al., 2012b). Following theoretical models of anxiety and empirical research, we explored the possibility that anxiety would be linked to early visual processing of threat (as indicated by increased amplitude of early ERP components; e.g., P1 and N170), as well as later elaborative processes (i.e., increased amplitude of the later LPP component). The inclusion of happy faces allowed some exploration of whether this pattern of neural activity would be specific to threat stimuli. We further anticipated that associations between trait and state anxiety symptoms and their interaction on threat processing would be clearer than those with depression. Based on theoretical models of depression (Armstrong and Olatunji, 2012) and previous literature (Foti et al., 2010; MacNamara et al., 2016) we predicted that depression symptoms would be associated with reduced/blunted LPP amplitudes to threat.

## Materials and Methods

### Participants

A hundred and eight children were approached via primary schools which agreed to forward a letter of information and consent to parents. Parents of 75 children gave consent for their child to participate in the study. Of those, pilot data from 5 children and data from 12 children (mean age = 7.50 years, *SD* = 1.20, age range 6–9 years) were excluded from analyses due to incomplete data and ERP artifacts. Complete ERP and behavioral data were available from 58 children (mean age = 8.80 years, *SD* = 1.60 years, age range 5.50–11.80, 37 boys). The study was approved by the Psychology Ethics Committee.

### Facial Expression Stimuli

Stimuli consisted of a standardized set of emotional facial expressions from two adult female models (Ekman and Friesen, 1976; Young et al., 2002) displaying prototypical anger and happiness and a neutral expression. We conducted a behavioral validation study of the facial stimuli in a separate community sample of 65 6–11-year-old children (mean age = 8.31 years, *SD* = 1.55, age range 6.00–10.75 years, 31 boys) recruited as above. Children viewed facial expressions one at a time (Angry, Happy, and Neutral, 12 trials per emotion), and were asked to identify the emotion in the face and press one of the three buttons with the labels ‘angry,’ ‘happy,’ or ‘neutral’ to indicate their response. The mean percentage of trials classified correctly was as follows: Angry: *M* = 94.10, *SD* = 14.56, Happy: *M* = 90.51, *SD* = 19.75, Neutral: *M* = 62.70, *SD* = 30.90. Accuracy was above-chance for all emotion types, with chance defined as 33.3% given the three response options. Age was significantly positively associated with accuracy for angry (*r* = 0.27, *p* < 0.05) but not happy (*r* = 0.08, *p* > 0.05) or neutral (*r* = 0.07, *p* > 0.05) faces. Accuracy for one emotion was correlated with accuracy for the other emotions (*r* > 0.27 and *p* < 0.05).

### Experimental Paradigm and Procedure

The experimental paradigm consisted of 180 experimental trials (60 trials per emotion type/30 trials per actor) presented in two blocks of 90 trials each. Children participated in 12 practice trials (four presentations of each emotion). Each trial began with the presentation of a central fixation cross (500 ms) followed by stimulus presentation (1000 ms) and a blank screen until participants responded, with a 1000 ms inter-trial interval. Emotion stimulus presentation was randomized across participants to prevent more than two faces of the same emotional category from appearing consecutively. Children viewed facial expressions one at a time (Angry, Happy, and Neutral), and were asked to identify the emotion in the face. Children were instructed to respond with their dominant hand and press one of three response buttons on a keyboard with the labels ‘angry,’ ‘happy,’ and ‘neutral’ to indicate their response. At the end of the session, children completed self-reported measures of anxiety and depression symptoms (see section “Symptoms of Trait and State Anxiety and Depression”).

### Symptoms of Trait and State Anxiety and Depression

Because the aim of the study was to examine individual differences in anxiety and depression, the sampling strategy employed aimed to identify the full range of clinical representation of children’s internalizing symptoms from no symptoms through to symptoms. The vast heterogeneity of internalizing symptoms imposed a continuous as opposed to categorical measure of child psychopathology. Self-report measures of trait anxiety and depression were taken via the DOMINIC (see Valla et al., 2000 for details on predictive validity), a DSM-IV based pictorial interview designed to assess a range of current psychiatric symptoms in 6- to 11-year-old children. In the DOMINIC, items are presented in the form of an interview via pictures accompanied by questions read to the children. The pictures illustrate the emotional and behavioral content of the DSM-IV (American Psychiatric Association, 2000) Axis I symptomatology. We used the generalized anxiety (14 items; ‘Do you worry a lot about not having friends?’) and depression (18 items; ‘Do you often feel like crying?’) scales. For each sentence there was a picture that described the character (DOMINIC) in the picture. Test–retest reliability for the DOMINIC is satisfactory with Kappa ranging from 0.40 to 0.70 (Valla et al., 2000). Cronbach’s alpha for child-reported anxiety and depression in the present study was >0.70 for both scales. Questions require a ‘yes’ (score 1) or ‘no’ (score 0) answer to create a total score with possible ranges of 0–14 and 0–18 for anxiety and depression, respectively. Parent-reports of symptoms were also collected using the DOMINIC but not included in analyses due to low reliability (for both scales Cronbach’s alpha < 0.20).

In addition, we asked children to report symptoms of state anxiety using the state anxiety questions from the State-Trait Anxiety Inventory for Children (STAIC; Spielberger, 1973). This consists of a 20-item 4-Likert-type scale (e.g., ‘I feel tense’) scored from (1) not at all, (2) somewhat, (3) moderately so and (4) very much so to generate a score range of 20–80 (α = 0.90 in the current sample).

### Electrophysiological Recording and Processing

**FIGURE 1:**
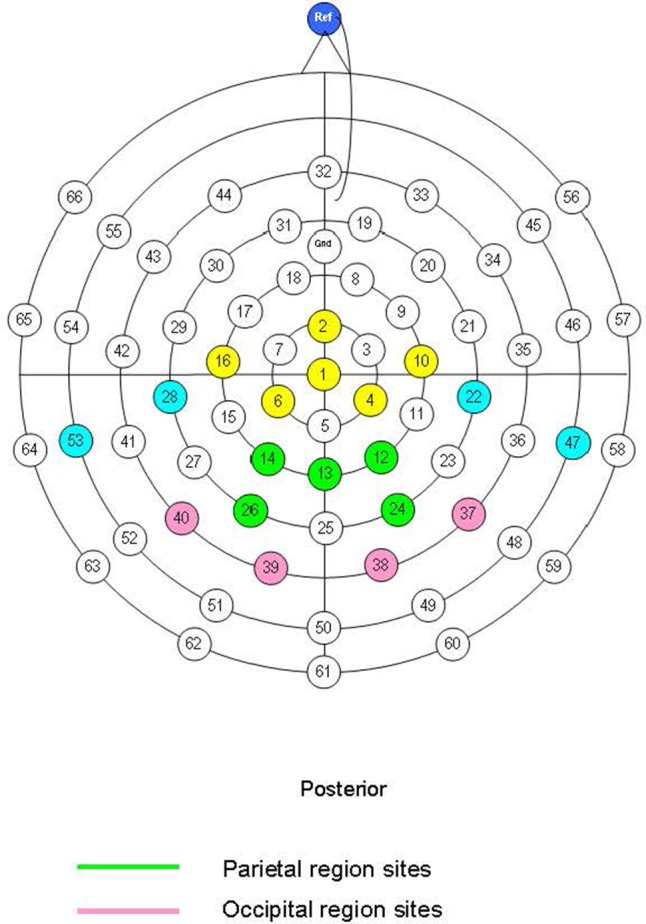
Montage with 19 sites used in EEG recording highlighted in yellow, blue, green, and pink. Sites used in analyses highlighted in green and pink for the parietal and occipital regions, respectively.

Electroencephalographic (EEG) data were recorded from an electrode cap (Easycap, Herrsching, Germany) containing 66 equidistant silver/silver chloride (Ag/AgCl) electrodes using Neuroscan Synamps^2^ 70 channel EEG system. Cap electrodes were referenced to the nose. The EEG data were sampled at 250 Hz with a band pass filter at 0.1–70 Hz and recorded from 19 sites. The equidistant montage with the sites used in EEG recording and analyses is shown in **Figure 1**. Analyses focused on nine sites at parietal (sites 12, 13, 14, 24, and 26) and occipital (sites 37, 38, 39, and 40) areas consistent with previous research (Batty and Taylor, 2006). A ground electrode was fitted midway between the electrode at the vertex and frontal site 32. Vertical electro-oculogram (vEOG) was recorded from two bipolar electrodes placed directly beneath the left and right eyes and two electrodes placed above the right and left eye included within the electrode cap. Impedances for vEOG, reference and cap electrodes were kept below 5 kω. The ERP epoch was defined as 100 ms pre-stimulus to 900 ms post-stimulus and was filtered with a low-pass filter down 48 dB at 32 Hz. An ocular artifact reduction procedure (Semlitsch et al., 1986) based on vEOG activity was used to remove the influence of blink and other eye movement; epochs were rejected if amplitudes exceeded ±150 μV at any EOG or scalp site included in analyses or if participants responded incorrectly. Average ERPs were calculated for each emotion type. A minimum of 20 artifact free epochs for each emotion type were used for calculating ERP averages. The mean and SD of the number of epochs for each emotion were as follows: Angry: *M* = 44.24, *SD* = 10.65, Happy: *M* = 44.17, *SD* = 10.70, Neutral: *M* = 42.90, *SD* = 11.46 (see Supplementary Material 1).

A mean amplitude method was followed for the P1 (110–200 ms), N170 (170–320 ms) and the LPP at parietal and occipital sites (see **Figure 1**). The LPP was observed in both early (LPP1: 430–520 ms) and late (LPP2: 520–610 ms) windows. Selection of this epoch length for the LPP was informed by previous literature in adults (Schupp et al., 2000) and children (Solomon et al., 2012; Kujawa et al., 2012a) indicating that the effects of emotion on the LPP are evident from 300 to 750 ms and become less stable at later windows. We also explored a later LPP window (610–900 ms) overlapping with the beginning of a negative slow wave but there were no significant main effects of emotion or emotion × group interaction effects for this window and these data are not reported further. Similarly, we explored the P300 amplitudes in a 340–430 ms window and found no significant main effects of emotion, emotion × laterality effects or associations with anxiety/depression. We chose to focus on 19 sites at parietal-occipital areas because the main components of interest in this study were maximal in posterior regions. This is consistent with previous literature showing that effects of emotion on LPP amplitudes become less stable in central and anterior scalp regions in children (Solomon et al., 2012). In addition, frontal channels are affected by ocular artifacts in children which can affect the number of clean epochs per condition and compromise the reliability of the ERPs. The mean amplitude was initially calculated for each individual site and then for each ERP component as a combined score for a number of electrode sites (‘scalp regions’-see **Figure 1**) to increase the reliability of measurement (see Dien and Santuzzi, 2005).

## Data Analysis

### Performance Data

Discrimination accuracy was computed for each target emotion using ‘hits’ (i.e., number of happy, angry, or neutral expressions classified correctly) according to the two-high-threshold model (Corwin, 1994; see Chronaki et al., 2012, 2013). Discrimination accuracy (Pr) is defined as sensitivity to discriminate a particular emotional expression and is given by the following equation: Pr = [(number of hits + 0.5)/(number of targets + 1)] – [(number of false alarms + 0.5)/(number of distractors + 1)] (Corwin, 1994). In other words, the Pr reflects the difference between the Hit rate and False Alarm rate, with values tending to 1, 0, and -1 for accuracy at better than chance, close to chance and worse than chance, respectively. Note that transformations are added in the above formulae (i.e., +0.5) to prevent divisions by zero. For example, in our task with 60 trials for each of the three conditions: angry, happy, and neutral, if a child classified 40 angry faces as angry but he/she also classified as angry 10 neutral faces and 10 happy faces, then his/her accuracy for angry faces would be: [(40 + 0.5)/(60 + 1)] - [(10 + 10 + 0.5)/(120 + 1)] = 0.49, suggesting that accuracy is better than chance. Spearman’s correlations examined the relationship between accuracy and trait anxiety symptoms (as measured by DOMINIC), depression and a composite score of ‘anxious/depressed’ symptoms (see below). To examine the effect of emotion type on accuracy, accuracy scores were entered in Friedman’s ANOVA with emotion as within-subject factor and paired Wilcoxon follow up tests. Mann–Whitney tests examined gender differences in accuracy for the three emotions.

### ERP Data

Preliminary analyses examined associations between child age and ERP amplitudes at each region. Independent-samples *t*-tests examined gender differences in ERP amplitudes. Preliminary analyses also examined the effect of (i) face model and (ii) task period on ERP amplitudes (see section “Preliminary ERP Analyses”). The trait anxiety and depression scales were highly intercorrelated (*r* = 0.80, *p* < 0.001), therefore, we created a composite score of ‘Anxious/Depressed Symptoms’ by summing anxiety and depression scores and we used the composite score in further analyses. For all analyses, the results were the same for combined or separate anxiety and depression scores. We created a High and Low ‘Anxious/Depressed Symptoms’ group (henceforth ‘Anx/Dep’ group) from a tertile split (i.e., the lowest and highest third of participant symptoms) of Anxious/Depressed symptom scores. We compared the ‘High’ (*n* = 19)’ and ‘Low’ (*n* = 19) groups from this tertile split in ERP analyses. The whole-sample correlations between ERPs and anxiety/depression scores were generally consistent with the pattern of results from the ANOVA analysis based on the high vs. low anxious/depressed groups. To facilitate the interpretation of the findings we report the high vs. low group analyses alongside the Pearson’s correlations in the whole sample between ERPs and anxiety/depression as continuous variables.

Repeated measures ANOVA was conducted including emotion (Angry, Happy, and Neutral) and laterality (Right and Left) as within-subjects factors and group (Low Anx/Dep and High Anx/Dep) as a between subjects factor to examine the main effects of emotion, group and group × emotion interaction effects on ERPs amplitude for each region (parietal and occipital), component (P1, N170, and LPP) and for the LPP only, time window (early-LPP1 and late-LPP2). Where there was a significant effect or interaction, these were followed-up with planned contrasts. In all analyses, planned contrasts compared angry and happy faces with neutral faces. We compared the right and left hemisphere after combining sites per region belonging to each hemisphere for the ERPs as follows: Right Parietal (sites 12, 24), Left Parietal (sites 14, 26), Right Occipital (sites 37, 38), Left Occipital (sites 39, 40). Finally, we ran hierarchical multiple regression analyses to explore whether trait anxiety/depression symptoms explained variance in LPP amplitude to angry vs. neutral faces above and beyond that explained by state anxiety symptoms. For these analyses we calculated scores for processing differences between angry and happy faces vs. neutral faces and where increased LPP scores indicated increased amplitudes for emotional vs. neutral faces.

## Results

The mean scores for self-report anxiety and depression from the DOMINIC were 5.00 (*SD* = 2.84) and 5.50 (*SD* = 3.40), respectively; 17.2% (scores > 8) and 13.8% (scores > 9) of the respective scores fell in the atypical (elevated) range (see Valla et al., 2000). The mean for the ‘Anxiety/Depression Symptoms’ composite score (see section “ERP Data”) was 10.60 (*SD* = 5.94). The mean state anxiety score was 26.70 (*SD* = 3.36) and was positively associated with the anxiety/depression composite score (*r* = 0.33, *p* = 0.012). The Low Anx/Dep group and High Anx/Dep group differed significantly in composite anxiety/depression scores [*F*(1,36) = 228.06, *p* < 0.001; Low: *M* = 4.20, *SD* = 1.68, High: *M* = 17.50, *SD* = 3.45] and state anxiety scores [*F*(1,36) = 8.66, *p* < 0.01; Low: *M* = 25.60, *SD* = 3.05, High: *M* = 28.60, *SD* = 3.10]. Child age was not associated with trait or state anxiety, depression or the composite score (*r*s < -0.17, *p*s > 0.18). There was no significant difference in anxiety or depression symptoms between males and females (*p*s > 0.05).

### Performance

Mean accuracy for all emotions are shown in **Table 1**. Accuracy values were not normally distributed due to ceiling effects, and could not be transformed, therefore, non-parametric tests were used. There was no significant gender difference in accuracy (*U* = 587, *Z* = -0.37, *p* > 0.05, *r* = -0.04). There was a tendency toward an effect of emotion on accuracy [χ^2^(2) = 5.45, *p* = 0.06] with higher accuracy scores for angry (*T* = 463, *p* < 0.05, *r* = -2.60) and happy (*T* = 511, *p* < 0.05, *r* = -2.50) compared to neutral faces. Spearman’s correlations between accuracy and trait anxiety, depression or the composite score were not significant (*r*s < -0.15, *p*s > 0.24). Spearman’s correlations between ERPs and accuracy showed that accuracy for happy faces was significantly correlated with occipital P100 to angry, happy and neutral faces (*r* > 0.30, *p* < 0.05).

**Table 1:** Mean percentage (SD) of trials classified correctly (in bold) and misattributions.

	Facial expression		
Identified as	Angry	Happy	Neutral
Angry	**92.27 (9.35)**	3.04 (4.90)	3.53 (4.50)
Happy	3.30 (4.60)	**92.18 (8.70)**	3.80 (5.70)
Neutral	5.48 (8.70)	3.82 (6.40)	**88.50 (14.30)**

### Preliminary ERP Analyses

**FIGURE 2:**
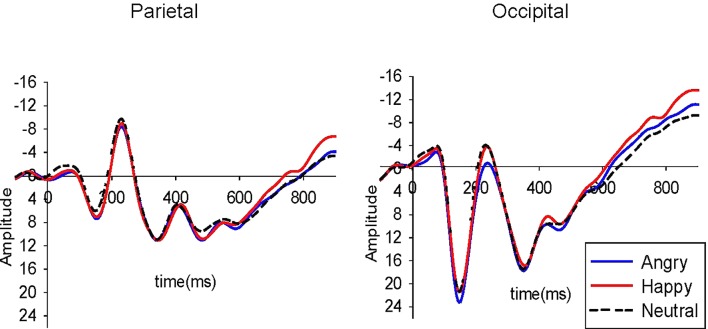
Grand mean ERPs to angry, happy, and neutral faces in the parietal and occipital region in the whole sample (*N* = 58). Amplitude (μV) and time (ms) are marked at the parietal and occipital regions with a pre-stimulus baseline of –100 ms. Scale is –16 to +25 μV.

**Figure 2** plots the grand mean averages to each emotion and region in the whole sample. Means and standard deviations for the ERP data are presented in **Table 2**. Child age was not associated with P1 or N170 amplitudes (*r*s < -0.08, *p*s > 0.55). Age was positively associated with parietal LPP amplitudes to happy and neutral faces for the early and late window (*r*s > 0.30, *p*s < 0.01). For this reason, LPP analyses for the early and late windows parietally were carried out with and without child age as a covariate. There was no significant difference in ERPs amplitude between males and females [*t*(56) < 1.7, *p* > 0.08].

**Table 2:** Means (SD) for the ERP components in the whole sample in the parietal and occipital region.

	Angry	Happy	Neutral
**Parietal**			
P100	9.74 (7.00)	9.90 (7.00)	9.08 (5.90)
N170	-11.35 (8.70)	-11.47 (9.27)	-11.87 (8.00)
LPP1	9.80 (8.75)	9.60 (9.27)	9.50 (10.24)
LPP2	9.00 (9.20)	8.55 (10.27)	8.52 (10.58)
**Occipital**			
P100	25.35 (9.20)	24.50 (10.40)	24.60 (8.86)
N170	-6.30 (9.60)	-7.80 (10.80)	-7.40 (9.76)
LPP1	9.45 (7.87)	8.70 (9.25)	9.90 (9.70)
LPP2	3.96 (8.77)	2.87 (10.20)	4.28 (10.27)

Preliminary analyses examined the effects of face model on ERPs and a time on task effect on ERP amplitudes to assess habituation of stimulus repetition. We conducted a repeated measures ANOVA with model (model 1 and model 2), task period (first half vs. second half) and emotion (Angry, Happy, and Neutral) as within-subject factors and ERP amplitude as the dependent measure. The results revealed no significant main or interaction effects (in all cases *F*s < 3.20 and *p*s > 0.08, see Supplementary Material 2 for details). Because there was no significant main effect of model or model × emotion interaction on ERPs amplitudes, ERP amplitudes to the two models were averaged for further analyses.

### ERP Analyses and Individual Differences

P1 and N170: The results showed a significant main effect of laterality on the P1 amplitudes in the parietal [*F*(1,36) = 19.73, *p* < 0.001, $\eta_{p}^{2}$ = 0.35] and occipital [*F*(1,36) = 18.20, *p* < 0.001, $\eta_{p}^{2}$ = 0.34] region. Planned contrasts showed larger P1 amplitudes in the right compared to the left hemisphere in the parietal [*F*(1,36) = 19.70, *p* = 0.002, $\eta_{p}^{2}$ = 0.24] and occipital [*F*(1,36) = 18.20, *p* < 0.001, $\eta_{p}^{2}$ = 0.33] region. In addition, there was a significant emotion × laterality × group interaction effect on occipital P1 [*F*(2,72) = 3.90, *p* = 0.025, $\eta_{p}^{2}$ = 0.09] but not on the parietal P1 [*F*(2,72) = 1.86, *p* = 0.16, $\eta_{p}^{2}$ = 0.05]. To break down this interaction for the occipital P1, contrasts were performed comparing angry with neutral and happy with neutral across each level of hemisphere (right vs. left) for the High Anx/Dep vs. Low Anx/Dep group. Planned contrasts revealed a significant difference between the two groups when comparing angry and neutral for the left compared to the right hemisphere [*F*(1,36) = 11.20, *p* = 0.002, $\eta_{p}^{2}$ = 0.24]. Specifically, there were significantly larger occipital P1 amplitudes to angry compared to neutral faces in the high compared to the low anxiety group for the left hemisphere. Finally, there was a significant laterality × group interaction effect on the parietal N170 amplitudes [*F*(1,36) = 7.20, *p* = 0.01, $\eta_{p}^{2}$ = 0.17]. To break down this interaction, contrasts were performed comparing right with left hemisphere for the High Anx/Dep and Low Anx/Dep groups. Planned contrasts revealed a significant difference between the two groups for the left compared to the right hemisphere [*F*(1,36) = 7.20, *p* = 0.01, $\eta_{p}^{2}$ = 0.17]. In particular, children with low anxiety/depression showed larger N170 amplitudes in the left compared to the right hemisphere, whereas children with high anxiety/depression showed larger N170 amplitudes in the right compared to the left hemisphere. When a Bonferroni correction was applied with an alpha level of 0.05/8 = 0.006 adopted, the emotion × laterality × group interaction effect on occipital P100 amplitudes remained significant. Results from the Pearson’s correlations showed significant positive associations between parietal and occipital P100 to angry-neutral amplitude difference scores and symptoms of state anxiety (*p*s > 0.30, *p* < 0.05). When a Bonferroni correction was applied with an alpha level of 0.05/12 = 0.004 adopted, the associations between the parietal (*r* = 0.38, *p* = 0.003) and occipital (*r* = 0.40, *p* = 0.003) P100 to angry–neutral amplitude difference scores and state anxiety remained significant. Associations between parietal (*r* = 0.33, *p* = 0.010) and occipital (*r* = 0.28, *p* = 0.034) N170 to angry–neutral amplitude difference scores and state anxiety were less strong and did not survive correction for multiple comparisons. Results are presented in **Table 3** (see also Supplementary Table S1 in Supplementary Material 3).

**Table 3:** Pearson correlations between child-report symptoms of negative affect (depression, trait and state anxiety) with Angry–Neutral and Happy–Neutral P1 and N170 amplitude difference scores in the parietal and occipital region in the whole sample (*n* = 58).

Symptoms	Angry–Neutral				Happy–Neutral			
	Parietal		Occipital		Parietal		Occipital	
	P1	N170	P1	N170	P1	N170	P1	N170
Trait anxiety	0.07	0.15	0.09	0.17	-0.05	-0.03	0.00	-0.07
State anxiety	0.38^∗∗^	0.33^∗∗^	0.40^∗∗^	0.28^∗^	0.26^∗^	0.06	0.26^∗^	0.18
Depression	0.12	0.17	0.15	0.10	0.07	0.05	0.05	-0.02

*^∗^p < 0.05, ^∗∗^p < 0.01, ^∗∗∗^p ≤ 0.001. Associations between the state × trait interaction term and ERPs were non-significant (ps > 0.08)*.

**FIGURE 3:**
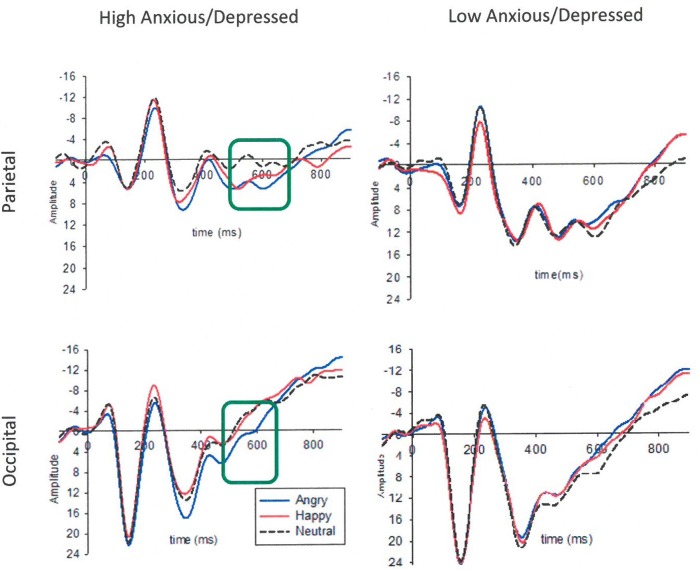
Grand mean ERPs to angry, happy and neutral faces in the High and Low Anxious/Depressed group using a tertile split. Larger LPP2 (520–610 ms) responses to the angry compared to neutral faces in the High Anxious/Depressed group in the parietal and occipital region are marked in the green box. Amplitude (μV) and time (ms) are marked with a pre-stimulus baseline of –100 ms. Scale is –15 to +26 μV.

**FIGURE 4:**
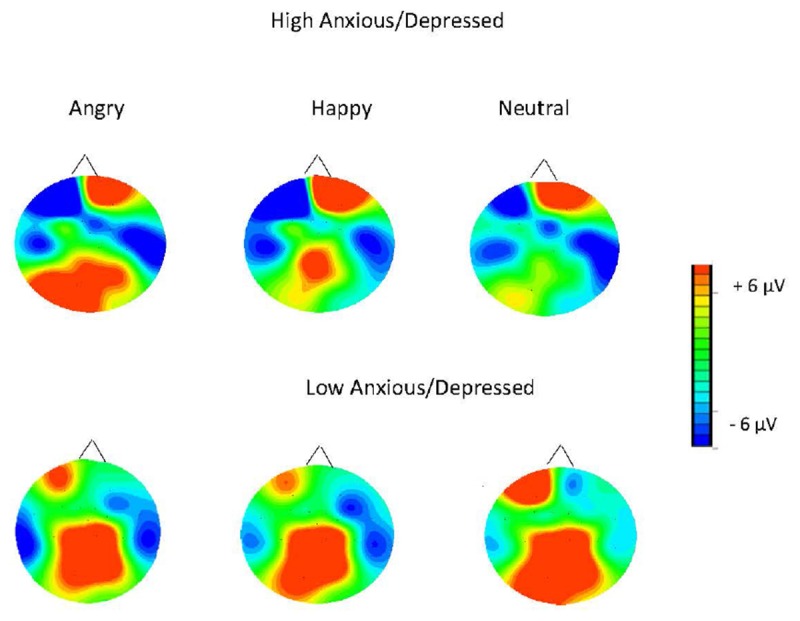
Topographic maps for the mean voltage distribution for the angry, happy, and neutral faces for the LPP2. Maps indicate larger posterior positivity (LPP2) in response to angry faces in the High Anxious/Depressed group compared to the Low Anxious/Depressed group. Scalp values represent the ends of the color scale in μV for the LPP2. Dark blue = negativity, red = positivity.

LPP: The results showed a significant emotion × group interaction effect on the occipital LPP1 [*F*(2,72) = 3.68, *p* = 0.030, $\eta_{p}^{2}$ = 0.09] and occipital LPP2 [*F*(2,72) = 4.85, *p* = 0.010, $\eta_{p}^{2}$ = 0.12] amplitudes. There was also a significant emotion x group interaction effect on the parietal LPP2 amplitudes [*F*(2,72) = 4.50, *p* = 0.014, $\eta_{p}^{2}$ = 0.10]. To break down these interactions, contrasts were performed comparing angry with neutral and happy with neutral for the High Anx/Dep vs. Low Anx/Dep group. Planned contrasts revealed a significant difference between the two groups when comparing angry and neutral for the occipital LPP1 [*F*(1,36) = 6.30, *p* = 0.017, $\eta_{p}^{2}$ = 0.15], occipital LPP2 [*F*(1,36) = 8.70, *p* = 0.006, $\eta_{p}^{2}$ = 0.19] and parietal LPP2 [*F*(1,36) = 8.40, *p* = 0.006, $\eta_{p}^{2}$ = 0.18]. Specifically, the LPP amplitudes were significantly larger to angry compared to neutral faces in the high Anx/Dep group compared to the Low Anx/Dep group (see **Table 4**). Results are presented in **Figures 3**, **4**. In addition, there was an emotion × laterality interaction effect on the LPP2 in the occipital [*F*(2,72) = 6.70, *p* = 0.002, $\eta_{p}^{2}$ = 0.16] and parietal [*F*(2,71) = 8.20, *p* = 0.001, $\eta_{p}^{2}$ = 0.18] region. To break down this interaction, contrasts were performed comparing angry with neutral and happy with neutral across each level of hemisphere (right vs. left). Planned contrasts revealed a significant difference between the two hemispheres for angry compared to neutral for the LPP2 in the occipital [*F*(1,36) = 4.70, *p* = 0.037, $\eta_{p}^{2}$ = 0.10] and parietal [*F*(1,36) = 7.26, *p* = 0.010, $\eta_{p}^{2}$ = 0.17] region. Specifically, the occipital and parietal LPP2 amplitudes were significantly larger to angry compared to neutral faces for the right compared to the left hemisphere.

**Table 4:** Summary of 3 emotion (angry, happy, and neutral) × 2 Anxiety/Depression group (High and Low) effects on LPP1 and LPP2 amplitudes at the parietal and occipital region.

	Contrast	Details	*F*-value	Significance
**Parietal**				
LPP1	Angry vs. Neutral	High Anx/Dep:7.30 vs. 3.60	4.45	0.040
		Low Anx/Dep:11.20 vs. 12.70		
LPP2	Angry vs. Neutral	High Anx/Dep: 6.80 vs. 2.70	8.40	0.006
		Low Anx/Dep: 9.20 vs. 11.45		
**Occipital**				
LPP1	Angry vs. Neutral	High Anx/Dep:7.30 vs. 3.80	6.30	0.017
		Low Anx/Dep:10.00 vs. 12.50		
LPP2	Angry vs. Neutral	High Anx/Dep:2.50 vs. -0.98	8.70	0.006
		Low Anx/Dep :4.38 vs. 6.80		

*For parietal LPP1, although the contrast comparing angry with neutral in the two groups was significant the top level emotion × group interaction effect was not significant (p = 0.06).*

There was also a marginally significant emotion × laterality × group interaction effect on the occipital LPP2 amplitudes [*F*(2,72) = 3.08, *p* = 0.050, $\eta_{p}^{2}$ = 0.08]. Contrasts were used to break down this interaction. The first contrast compared occipital LPP2 scores of the high and low anxiety group for angry compared to neutral faces and right compared to left hemisphere. This contrast revealed a significant difference between the high and low anxiety group when comparing angry to neutral faces and right to the left hemisphere [*F*(1,36) = 4.38, *p* = 0.040, $\eta_{p}^{2}$ = 0.10]. This showed that for the left hemisphere occipital LPP2 amplitudes were higher to angry compared to neutral faces for the high anxiety group, whereas for the low anxiety group LPP2 amplitudes were higher to neutral, compared to angry faces. For the right hemisphere, however, there was no difference between the groups in LPP2 amplitude to angry vs. neutral faces (see **Figure 6**). The second contrast revealed a non-significant difference between the two groups when comparing happy to neutral faces when the right hemisphere was compared to the left hemisphere [*F*(1,36) = 0.90, *p* = 0.75, $\eta_{p}^{2}$ = 0.003].

**FIGURE 5:**
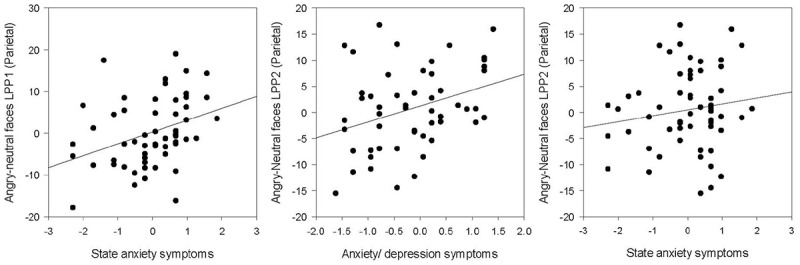
Associations between the LPP1 and LPP2 amplitudes in the parietal region for trait anxiety/depression and state anxiety.

When a Bonferroni correction was applied with an alpha level of 0.05/8 = 0.006 adopted, only the effects related to the LPP2 remained significant. Results from the Pearson’s correlations showed significant positive associations between parietal and occipital LPP to angry-neutral amplitude difference scores and symptoms of state anxiety, trait anxiety and depression (*p*s > 0.38, *p* < 0.01). Results are presented in **Figure 5**. When a Bonferroni correction was applied with an alpha level of 0.05/12 = 0.004 adopted, the associations between the occipital LPP2 to angry-neutral amplitude difference scores and trait anxiety (*r* = 0.40, *p* = 0.002) and depression (*r* = 0.39, *p* = 0.002) remained significant. Associations between the parietal LPP2 to angry-neutral amplitude difference scores and state anxiety (*r* = 0.39, *p* = 0.003) and depression (*r* = 0.40, *p* = 0.002) also remained significant. However, associations between parietal and occipital LPP1 to angry-neutral amplitude difference scores and anxiety/depression did not remain significant (*p*s > 0.009). Results are presented in **Table 5** (see also Supplementary Table S2 in Supplementary Material 3).

**Table 5:** Pearson correlations between child-report symptoms of negative affect (depression, trait and state anxiety) with Angry–Neutral and Happy–Neutral LPP amplitude difference scores in the parietal and occipital region in the whole sample (*n* = 58).

Symptoms	Angry–Neutral				Happy–Neutral			
	Parietal		Occipital		Parietal		Occipital	
	LPP1	LPP2	LPP1	LPP2	LPP1	LPP2	LPP1	LPP2
Trait anxiety	0.20	0.33^∗^	0.29^∗^	0.40^∗∗^	0.19	0.22	0.19	0.19
State anxiety	0.34^∗∗^	0.39^∗∗^	0.35^∗∗^	0.36^∗∗^	0.18	0.15	0.18	0.13
Depression	0.27^∗^	0.40^∗∗^	0.29^∗^	0.39^∗∗^	0.28^∗^	0.21	0.17	0.11

*^∗^p < 0.05, ^∗∗^p < 0.01, ^∗∗∗^p ≤ 0.001. Associations between the state x trait interaction term and ERPs were non-significant (ps > 0.08)*.

**FIGURE 6:**
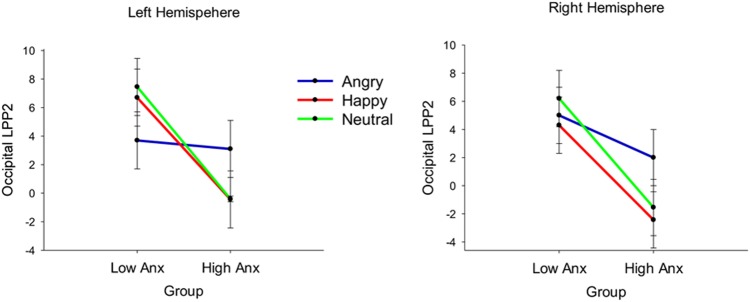
Line graph with error bars showing the occipital LPP2 amplitudes per emotion and hemisphere in the two groups. Emotion × laterality × group interaction shows that for the left hemisphere occipital LPP2 amplitudes were higher to angry compared to neutral faces for the high anxiety group, whereas for the low anxiety group LPP2 amplitudes were higher to neutral, compared to angry faces.

Furthermore, hierarchical multiple regression analyses were run to directly test whether trait anxiety/depression symptoms explained variance in LPP amplitude to angry vs. neutral faces above and beyond that explained by state anxiety symptoms. The parietal and occipital LPP amplitudes were entered as the outcome variable. Predictor variables included state anxiety entered in the first block, the trait anxiety/depression composite score and the interaction with state anxiety in the second block^1^. The results showed that state anxiety explained 13% of the variance in the occipital LPP2 amplitudes to angry relative to neutral faces [*F*(1,56) = 8.17, *p* < 0.01, *R*^2^ = 0.13, *R*^2^_Adjusted_ = 0.11]. When trait anxiety/depression composite score and the state × trait anxiety interaction term were added as predictors (model 2), this increased to 26% of the total variance [*F*(3,54) = 6.48, *p* = 0.001, *R*^2^ = 0.26, *R*^2^_Adjusted_ = 0.22]. In model 2, there was a significant association between trait anxiety/depression composite score and the occipital LPP2 to angry vs. neutral face (*p* < 0.05), suggesting that trait anxiety/depression explained variance in the LPP amplitudes to angry vs. neutral faces above and beyond that explained by state anxiety. No other association with angry or happy vs. neutral scores was significant; see **Table 6** and **Figures 3**–**6**.

**Table 6:** Hierarchical multiple regression examining the independent contribution of state anxiety, trait anxiety/depression composite score and state × trait anxiety interaction on the occipital LPP2 to angry vs. neutral faces.

	Occipital LPP2			
	*b*	*SEB*	β	*p*
**Model 1**				
State anxiety	0.80	0.27	0.35	0.006
**Model 2**				
State anxiety	0.50	0.27	0.24	0.056
Trait anxiety/depression	0.37	0.16	0.30	0.020
State × trait anxiety	1.67	1.03	0.20	0.110

*R^2^ = 0.13 for Model 1: R^2^ = 0.26 for Model 2, ΔR^2^ = 0.13 for Model 2.*

## Discussion

The current study examined the association between the neural processing of angry and happy (vs. neutral) facial stimuli with child report symptoms of trait and state anxiety and depression in 6–11 year old children. The results showed that the P1 was larger to anger than neutral faces in the left hemisphere, though early components (P1, N170) were not strongly associated with anxiety and depression symptoms. In contrast, anxiety/depression symptoms were positively associated with LPP amplitudes to angry (vs. neutral) faces. Finally, the differences between LPP amplitudes for angry vs. neutral faces were independently associated with measures of state and trait anxiety/depression symptoms.

The early components (P1, N170) did not show sensitivity to facial emotion in our study, consistent with previous research in children (Todd et al., 2008; Dennis et al., 2009). The LPP to emotional and neutral stimuli emerged in similar time windows (∼350 to 750 ms) and scalp regions as reported in previous studies (e.g., Schupp et al., 2000; Hajcak and Dennis, 2009).

Children with high anxiety/depression displayed larger LPP amplitudes to angry (vs. neutral) faces. This effect was clearest for the second LPP time window in parietal and occipital regions. These results are consistent with previous research showing increased processing of angry compared to neutral stimuli (reflected by the LPP) in highly anxious adults (Holmes et al., 2008) and children during reappraisal tasks (DeCicco et al., 2012). Finally, our study found larger P100 amplitudes to angry (vs. neutral) faces in the left hemisphere in the high compared to the low anxiety group, possibly suggesting greater early, sensory processing of threat in anxiety/depression. Findings extend previous work in a community sample of adults with high trait anxiety who have shown larger amplitudes of early latency components (e.g., P2) when viewing angry faces (Bar-Haim et al., 2005).

The stronger effects in the current study related to the angry compared to neutral faces. Neutral expressions are argued to be highly ambiguous and potentially threatening for children (Melfsen and Florin, 2002; Yoon and Zinbarg, 2007). This interpretation is consistent with the lower accuracy scores for neutral compared to angry and happy expressions in our study. Similar research has shown that children displayed greater amygdala activation in response to neutral than fearful faces (Thomas et al., 2001). In contrast, adults showed increased left amygdala activity for fearful faces relative to neutral faces in the same study. Findings highlight the need to address the specificity of differential neural responses by employing positive stimuli and different types of negative stimuli (e.g., anger and fear) in future studies.

Our study showed differential effects of anxiety/depression symptoms for early (P100/N70) compared to late (LPP) components. This pattern of results may reflect differential functional locus of anxiety effects in evaluative compared to perceptual domains of processing social signals of threat. While the present study did not support broader links between anxiety and attentional biases toward threat at early stages of perceptual processing it is possible that neural patterns of early biases observed in adults (Bar-Haim et al., 2005; Eldar et al., 2010) are not developmentally evident in middle childhood. Alternatively, recent conceptualizations of attention in anxiety suggest that attention to threat in anxiety is clearest when presenting stimuli that compete for attention and at relatively short stimulus presentation durations (review by Richards et al., 2014). This raises the possibility that face categorization tasks (and when presented for longer periods) are more sensitive to evaluative cognitive processes in anxiety. The exact mechanisms that underlie the neural development of early processing biases toward threat and sensitivity of individual differences to different experimental paradigms in childhood anxiety require further investigation.

In addition, this study extended previous research to demonstrate that both elevated state and trait anxiety symptoms contributed independently to variation in the LPP amplitude to angry compared to neutral faces. These findings are consistent with the proposition that state anxiety and trait anxiety can contribute independently to attentional biases (Mogg et al., 1990). Specifically the difference between amplitudes to angry vs. neutral faces was positively associated with state anxiety in the first LPP time window and subsequently with both increased trait anxiety/depression symptoms and state anxiety in the second LPP time window across parietal and occipital regions. The findings support cognitive models of attention to threat in anxiety (Mogg and Bradley, 1998) and demonstrate that state and trait anxiety contribute independently to the neural response to threat during childhood. This pattern of results was observed with angry, but not happy stimuli suggesting that the neural development of information processing biases in childhood anxiety is specific to threat rather than emotionally arousing stimuli in general. It is important to note that although ERPs can show differences between groups of subjects that can elucidate mechanisms of development of developmental disorders, because of their variability ERPs are less helpful in determining whether an individual child is developing abnormally (Picton and Taylor, 2007). One should consider carefully the variability of ERPs in terms of latency and amplitude in groups of children and average ERPs across participants (Picton et al., 2000).

Several studies have explored the role of state anxiety on attentional biases to threat stimuli (e.g., Fox et al., 2001; Quigley et al., 2012; Nelson et al., 2014). Fox et al. (2001) found that individuals with elevated state anxiety showed difficulties disengaging from threatening (angry) faces compared to those with low state anxiety. More recent research has explored attentional processes to simultaneously presented emotional (threat or happy) vs. neutral images in state anxiety before an attention task (baseline state) and after experiencing an anxious mood manipulation (elevated state; Quigley et al., 2012). They found that individuals with increased baseline and elevated symptoms of state anxiety viewed threat (vs. neutral) images for a greater proportion of the time. Consistent with the findings of Fox et al. (2001), Quigley et al. (2012) showed that elevated state anxiety was also linked to increased time spent looking at threat images on first fixation (indicating some difficulties with disengagement). Quigley et al. (2012) argued that these findings link to Bower’s (1981) proposition that individuals are predisposed to attend to and recall “mood-congruent” (p.138) information. Bower’s (1981) emotion network theory highlights emotion-relevant attentional processes in chronic negative emotional states, like anxiety. Quigley et al. (2012) further highlight that these results support brain imaging studies that have found links between state anxiety with increased amygdala activation to fearful stimuli (e.g., Bishop et al., 2004). Consistently, a recent eye movement study showed that children aged 9–11 years who reported elevated symptoms of neuroticism showed increased latencies to move their eyes away from angry faces to identify a target stimulus, supporting difficulties with disengagement (Pavlou et al., 2016).

Importantly, the above effects were not specific to anxiety symptoms but also generalized to depression symptoms, suggesting common neural substrates of information processing biases in childhood internalizing symptomatology. Recent work suggests that anxiety and depression may have opposing associations with the LPP; while anxiety is associated with *enhanced* LPP to threat, depression is associated with *reduced* LPP to threat (MacNamara et al., 2016). It is further suggested that blunted/reduced LPP emotional response in depression persists even when controlling for the presence of generalized anxiety (MacNamara et al., 2016). Our results support a pattern of *enhanced* LPP to threat linked to *both* anxiety and depression symptoms, suggesting that anxiety and depression may share overlapping cortical mechanisms to threat biases. This similarity in underlying emotional brain circuits may explain the similarity of behavioral manifestations of symptoms in the two conditions. However, because child report symptoms of anxiety and depression were highly inter-correlated in this study, it was not possible to disentangle their independent effects. Future studies should employ pure and comorbid groups of childhood anxiety and depression to understand key emotional processes associated with childhood internalizing psychopathology and aid the identification of causal mechanisms and treatment targets (Kring, 2010).

In summary, this study extends previous research to identify neurobiological markers of attentional biases in children with state and trait anxiety and depression symptoms. The results are consistent with theoretical models of anxiety to highlight increased processing of threat in individuals with elevated trait anxiety (e.g., Mogg and Bradley, 1998; Bar-Haim et al., 2007). The findings are therefore relevant to the development of interventions that focus on emotion regulation and attentional control in this group of children. Consistent with the notion of mood congruent processing (Bower, 1981), our results also indicate that when children report temporary feelings of anxiety, as expressed in state anxiety measures, biases for the processing of threatening information are also evident. A significant limitation in the study is the high correlations between anxiety and depression; however, this is a common problem in the literature (Holmes et al., 2008). The conclusions are also limited by the properties of the stimuli used. Complex emotional images may be more effective in eliciting larger LPPs to emotional compared to neutral stimuli in children. Future studies would also benefit from a more diverse stimulus set of female models. Moreover, effects of anxiety and depression on the LPP were observed in a small time window (520–610 ms) and although we also explored a later LPP window (610–900 ms) we did not observe effects of anxiety on this late LPP. Future research should replicate the present findings in clinical samples of children with anxiety and depression, taking into account measures of state anxiety. Future research should also aim to employ larger sample sizes and examine attention bias using dot-probe tasks which can more readily examine potential behavioral biases. Despite the above limitations, the present study provided novel evidence that neural abnormalities underlying the processing of threat-related stimuli in childhood state and trait anxiety/depression occur at later, more evaluative and effortful processing stages rather than earlier, perceptual processing stages.

## Author Contributions

All authors listed have made a substantial, direct and intellectual contribution to the work, and approved it for publication.

## Conflict of Interest Statement

The authors declare that the research was conducted in the absence of any commercial or financial relationships that could be construed as a potential conflict of interest.

## Footnotes

1 ERPs for neutral faces were subtracted from those for angry faces to create a difference score. Higher scores indicated greater amplitudes for angry relative to neutral faces. This was repeated for the happy faces. The interaction term between state and trait anxiety was created by multiplying centered variables.
