# Supplementary material for: The Moderating Effect of Self-Reported State and Trait Anxiety on the Late Positive Potential to Emotional Faces in 6–11-Year-Old Children
Source: Front Psychol. 2018 Feb 20;9:125. doi: 10.3389/fpsyg.2018.00125 (PMC5826320; doi:10.3389/fpsyg.2018.00125)
Supplement: Supplementary file 1 [file Table_3.pdf]

Supplementary material 3

Table S1. Pearson correlations between child-report symptoms of negative affect (depression, trait and state anxiety) with Angry-Neutral and Happy-Neutral P100 and N170 amplitude difference scores in the parietal and occipital region in the right and left hemisphere in the whole sample (n=58).

	Angry-Neutral				Happy-Neutral			
	Parietal		Occipital		Parietal		Occipital	
Symptoms	P1	N170	P1	N170	P1	N170	P1	N170
**Right Hemisphere**								
Trait anxiety	.02	.15	.00	.14	-.02	.01	-.04	.12
State anxiety	**.35****	**.33***	**.32***	**.23**	.22	.05	.22	-.06
Depression	.07	.14	.05	.04	.07	.07	.04	-.05
**Left Hemisphere**								
Trait anxiety	.10	.14	**.26***	.18	-.06	.09	.04	-.08
State anxiety	**.38****	**.32***	**.40****	**.30***	**.29***	.09	**.30***	.23
Depression	.17	.16	**.25***	.15	.08	.02	.06	.00

Note:*p <.05, **p< .01, ***p ⩽.001. Associations between the state x trait interaction term and ERPs were non-significant (ps >.06)

Table S2. Pearson correlations between child-report symptoms of negative affect (depression, trait and state anxiety) with Angry-Neutral and Happy-Neutral LPP amplitude difference scores in the parietal and occipital region in the right and left hemisphere in the whole sample (n=58).

	Angry-Neutral				Happy-Neutral			
	Parietal		Occipital		Parietal		Occipital	
Symptoms	LPP1	LPP2	LPP1	LPP2	LPP1	LPP2	LPP1	LPP2
**Right Hemisphere**								
Trait anxiety	.18	**.32***	.23	**.32***	.20	.21	.23	.18
State anxiety	**.30***	**.37****	**.31***	**.35****	.16	.18	.18	.20
Depression	.22	**.38****	.23	**.34****	**.26***	.20	.23	.16
**Left Hemisphere**								
Trait anxiety	.22	**.34***	**.34****	**.45****	.17	.20	.16	.17
State anxiety	**.34****	**.35****	**.38****	**.34****	.18	.09	.17	.05
Depression	**.28***	**.37****	**.35****	**.43****	.25	.15	.13	.05

Note:*p <.05, **p< .01, ***p <.001. Associations between the state x trait interaction term and ERPs were non-significant (ps >.08)
