# Supplementary material for: The Moderating Effect of Self-Reported State and Trait Anxiety on the Late Positive Potential to Emotional Faces in 6–11-Year-Old Children
Source: Front Psychol. 2018 Feb 20;9:125. doi: 10.3389/fpsyg.2018.00125 (PMC5826320; doi:10.3389/fpsyg.2018.00125)
Supplement: Supplementary file 2 [file Data_Sheet_1.pdf]

Supplementary material 1

There was no significant effect of emotion condition on the number of correct and artifact free epochs (F (1, 57) = 1.90, p>.16). We examined potential effects of age on artifact and trial rejection by dividing our sample into younger (5.50-8.70 years) and older (8.75-11.80 years) children using a median split. There was a significant effect of age on the number of correct and artifact free trials included in analyses for angry (F (1, 57) = 12.77, p<.01), happy (F (1, 57) = 9.84, p<.01) and neutral (F (1, 57) = 13.60, p<.01) faces with fewer trials for younger children (Angry: M=39.68, SD=11.09, Happy: M=40.06, SD=10.86, Neutral: M=37.80, SD=11.90) compared to older children (Angry: M=48.80, SD=8.07, Happy: M=48.27, SD=8.95, Neutral: M=47.86, SD=8.47). Age effects in our study are similar to age effects reported in other ERP studies utilising facial stimuli with children of similar ages (Vlamings et al., 2010). Child symptoms were not correlated with the number of correct and artifact-free trials (r >.-24, p <.08).

References for Supplementary material 1

Vlamings, P. H. J. M., Jonkman, L. M. & Kemner, C. (2010). An Eye for Detail: An Event-Related Potential Study of the Rapid Processing of Fearful Facial Expressions in Children. *Child Development, 81*(4), 1304-131
