# Supplementary material for: The Moderating Effect of Self-Reported State and Trait Anxiety on the Late Positive Potential to Emotional Faces in 6–11-Year-Old Children
Source: Front Psychol. 2018 Feb 20;9:125. doi: 10.3389/fpsyg.2018.00125 (PMC5826320; doi:10.3389/fpsyg.2018.00125)
Supplement: Supplementary file 3 [file Data_Sheet_2.pdf]

Supplementary material 2

The repeated measures ANOVA with model (model 1, model 2), task period (first half vs. second half) and emotion (Angry, Happy, Neutral) as within-subject factors and ERP amplitude as the dependent measure showed no significant main or interaction effects (in all cases Fs < 3.20 and *ps* >.08). Specifically, for the occipital P100 there was no significant main effect of task period ($F(1, 43) = .37$, $p = .50$, $\eta^2_p = .009$), model ($F(1, 43) = .08$, $p = .74$, $\eta^2_p = .002$) or interaction effect ($F(1, 43) < 0.5$, $p > .60$, $\eta^2_p < .01$) on amplitude. Similarly, for the occipital N170 there was no significant main effect of task period ($F(1, 43) = .002$, $p = .96$, $\eta^2_p = .001$), model ($F(1, 43) = .13$, $p = .70$, $\eta^2_p = .003$) or interaction effect ($F(1, 43) < 0.2$, $p > .80$, $\eta^2_p < .007$) on amplitude. The pattern of results was the same for the parietal P1 and N170 amplitudes. For the occipital LPP1 there was no significant main effect of task period ($F(1, 43) = .70$, $p = .40$, $\eta^2_p = .01$), model ($F(1, 43) = .24$, $p = .62$, $\eta^2_p = .01$) and there was a tendency for a model x emotion interaction effect ($F(1, 43) = 3.00$, $p = .08$, $\eta^2_p = .06$) on amplitude, indicating a trend for larger LPP1 amplitudes for angry versus neutral for model 1 compared to model 2. This pattern of results was similar for the occipital LPP2. For the parietal LPP1 there was no significant main effect of task period ($F(1, 43) = 1.00$, $p = .32$, $\eta^2_p = .020$), model ($F(1, 43) = .38$, $p = .54$, $\eta^2_p = .009$) or interaction effect ($F(1, 43) < 2.80$, $p > 1.00$, $\eta^2_p < .06$) on amplitude. This pattern of results was similar for the parietal LPP2.
